# Species distribution models throughout the invasion history of Palmer amaranth predict regions at risk of future invasion and reveal challenges with modeling rapidly shifting geographic ranges

**DOI:** 10.1038/s41598-018-38054-9

**Published:** 2019-02-20

**Authors:** Ryan D. Briscoe Runquist, Thomas Lake, Peter Tiffin, David A. Moeller

**Affiliations:** University of Minnesota, Department of Plant and Microbial Biology, 1479 Gortner Avenue, St. Paul, 55108 MN, USA

## Abstract

Palmer amaranth (*Amaranthus palmeri*) is an annual plant native to the desert Southwest of the United States and Mexico and has become invasive and caused large economic losses across much of the United States. In order to examine the temporal and spatial dynamics of past invasion, and to predict future invasion, we developed a broad array of species distribution models (SDMs). In particular, we constructed sequential SDMs throughout the invasion history and asked how well those predicted future invasion (1970 to present). We showed that invasion occurred from a restricted set of environments in the native range to a diverse set in the invaded range. Spatial autocorrelation analyses indicated that rapid range expansion was facilitated by stochastic, long-distance dispersal events. Regardless of SDM approach, all SDMs built using datasets from early in the invasion (1970–2010) performed poorly and failed to predict most of the current invaded range. Together, these results suggest that climate is unlikely to have influenced early stages of range expansion. SDMs that incorporated data from the most recent sampling (2011–2017) performed considerably better, predicted high suitability in regions that have recently become invaded, and identified mean annual temperature as a key factor limiting northward range expansion. Under future climates, models predicted both further northward range expansion and significantly increased suitability across large portions of the U.S. Overall, our results indicate significant challenges for SDMs of invasive species far from climate equilibrium. However, our models based on recent data make more robust predictions for northward range expansion of *A. palmeri* with climate change.

## Introduction

Invasive species are marked by rapid range expansion and dramatic population growth that negatively affects communities and ecosystems outside of their historical range^1^. Because invasive species often cause considerable economic losses, land managers and conservation scientists are in need of tools to forecast invasion risk so that they can direct resources for prevention strategies and targeted surveillance operations^2–6^. Species distribution models (SDM) use species occurrence records and environmental data to build correlative models of habitat suitability and identify key environmental variables limiting range expansion^7–10^. For invasive species, SDMs can be a useful tool for identifying potential habitat requirements and environmental limitations of future range expansion^6,9,11,12^. Further, the models provide testable hypotheses that can be evaluated with field experiments and long-term observation of population dynamics. However, because invasive species often violate key assumptions of SDMs, we do not fully understand the extent to which they are reliable for predicting potential invasive species’ range expansion^9,13^. In this study, we leveraged detailed historical records for the rapid invasion of Palmer amaranth (*Amaranthus palmeri*) to assess if and how SDMs predict the explosive range expansion during the invasion process. Because *A. palmeri* is among the most problematic emerging threats to natural (e.g. prairies, grasslands) and agricultural ecosystems in the United States, accurate SDMs may be particularly valuable for preventing and mitigating future invasion.

Developing SDMs for invasive species can be considerably more challenging than for native species. SDMs operate under the assumption that a species is at equilibrium with the environment and that there are not large areas that are suitable but unoccupied; however, invasive species are, by definition, not at equilibrium^9^. SDM methods have been adapted to ameliorate some of the inherent problems of invasive species modelling but these methods can have differing effects on invasion projections and interpretation. For example, because environments (and correlations among environmental variables) may differ outside of the range compared to inside, modelers may retain more environmental variables to capture this variation^9,14^. In addition, it is unclear whether models built using invaders’ native ranges improve or hinder predictions of future range expansion^6,15–17^. Developing SDMs for invasive species can be especially problematic in cases where adaptation and/or admixture influence niche breadth over the course of invasion history^17–19^. In these cases, the genetic composition of populations, and thus the environmental factors that determine reproductive success, may differ between the native and invaded ranges. As a result, both ecological (dispersal limitation) and evolutionary factors (gene flow, adaptation) violate key assumptions of SDMs.

Although invaders often arrive from other continents^1,20^, they can also emerge within a single continent and exhibit rapid range expansion^21–24^. Similar to trans-continental invaders, within-continent invaders often exhibit explosive demographic expansions and can cause similarly disruptive ecosystem effects^22–24^. At the same time, these invaders may possess a unique subset of characteristics (e.g. association with anthropogenic change) and management challenges that are vital to document and model in order to develop effective management strategies^1,23,24^. Modelling within-continent invaders offers the opportunity to study how SDMs perform when an invasion occurs directly from a native range to adjacent ecosystems and across continuous environmental gradients. Investigations of native invasion can more readily distinguish among the alternative causes of sudden range expansion including climate, biotic interactions (e.g. competitive environment), or dispersal limitation^22–24^.

*Amaranthus palmeri* is an annual, dioecious plant with a relatively narrow native range that includes the desert Southwest of the United States (especially Arizona and New Mexico) and northwest Mexico (Sonora)^25^. Beginning in the mid 20^th^ century, its range rapidly expanded north and east with populations now found throughout most of the continental United States, except for New England, the Rocky Mountain region, and Pacific Northwest^25–27^. In the last two decades, *A. palmeri* has become one of the most economically-damaging weeds in the United States^28,29^. Invasion has occurred most commonly into restorations of natural habitat (i.e. conservation plantings) and agricultural fields via contaminated seed sources and agricultural equipment, respectively^28,30^. Crop yields in fields infested with *A. palmeri* may be reduced by more than half^31,32^ and yield losses have the potential to reach as high as four billion dollars annually in the mid-South alone^33^. Despite the enormous economic impact and continued range expansion, the environmental controls of its distribution and predictions for its future spread remain poorly resolved.

In this study, we examined the range expansion dynamics of *A. palmeri* from its historical to current range and used SDMs to predict the distribution of suitable habitat across North America throughout the invasion process and under future climates. To determine whether SDMs would have been successful if implemented at early versus late stages of the invasion, we took advantage of the detailed record of invasion and abundant occurrence dataset to construct SDMs at five time points in the invasion process. Because rapidly-expanding invasive species can be challenging to model, we used a broad array of modeling approaches (native + invaded range vs. invaded range only, Maxent vs. boosted regression trees, alternative climate datasets, downsampling overreported areas, etc.) to determine their consequences for model accuracy and discrimination ability under both current and future climates. We also determined which environmental factors most influenced projected habitat suitability and potentially limit range expansion into unoccupied regions. Finally, we examined the contribution of stochastic long-distance dispersal to range expansion using spatial autocorrelation analyses.

## Results

### Niche breadth and overlap between the native versus invaded ranges

We characterized the climate niche breadth of *A. palmeri* and quantified the extent to which the climate niche has shifted and/or expanded in the invaded range. We used an approach that first involves principal components analysis (Fig. S1) and then accounts for the frequency of occurrence records in environmental space and the frequency of environments^34^.

**Figure 1:**
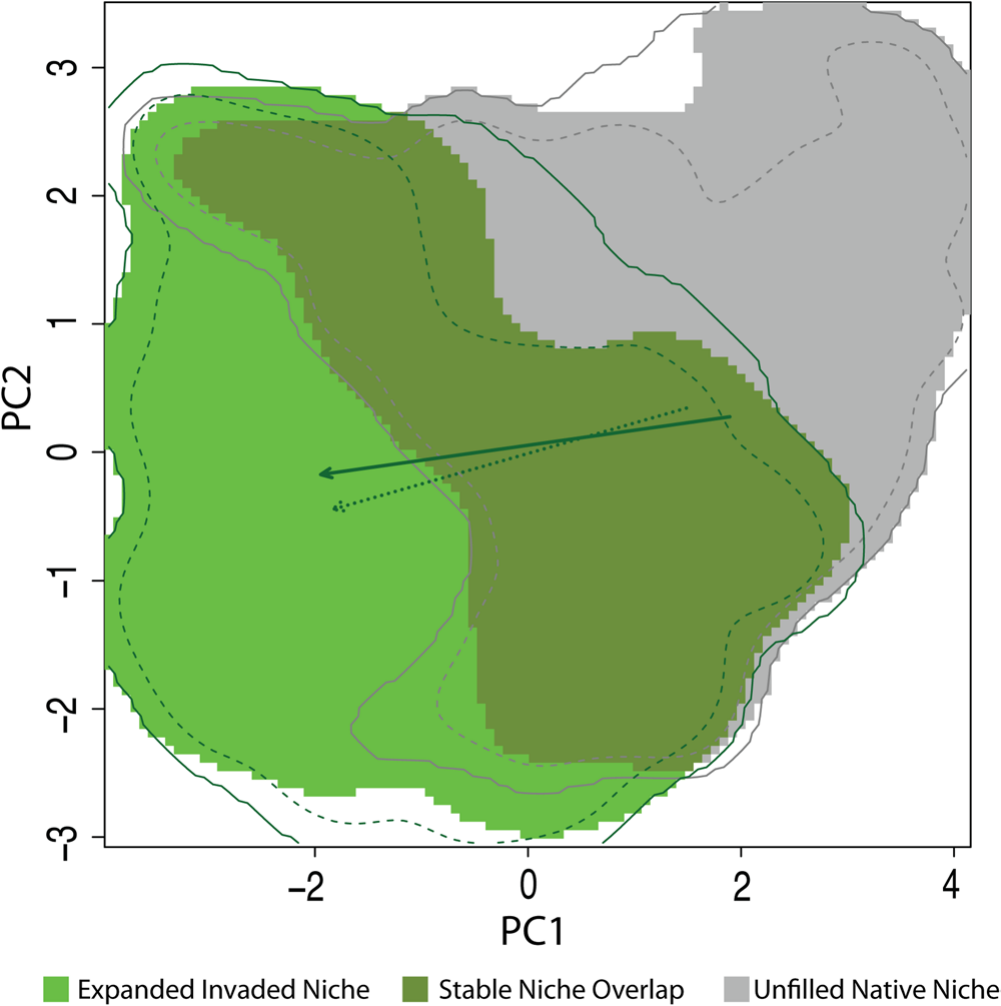
Niche breadth and shift of native versus invaded niche. Axes are principal components of CliMond variables included in the model. The total extent of the background environment in the native region is outlined in grey (solid = total niche space; dashed = 90% of extent). The total extent of the background environment in the invaded region is outlined in dark green (solid = total niche space; dashed = 90% of extent). The environmental space unique to the native niche is shown in gray (9.1% weighted loss of the original niche), the area of environmental space shared between the native and invaded niche is shown in olive-green (87% of the total weighted invaded niche), and the region of environmental space newly occupied by the invaded niche is shown in green (i.e. niche expansion; 13% if the total weighted invaded niche). The arrows represent the shift in the weighted centroid of occurrences from native to invaded niche (dark solid arrow), and the shift in the center of environmental space (lighter dashed arrow). The weighted niche overlap, calculated using Schoener’s *D*, was 0.32.

The invaded range represented both a niche shift (change in centroid) and niche expansion (increase in extent) based on principal components of both CliMond (Fig. 1) and PRISM climate data (Fig. S2). This niche shift and expansion was also apparent along individual environmental variable axes (Fig. S3). Further, niche shift and expansion were evident from niche similarly tests^34^, which indicated that in the invaded range, the environments in which *A. palmeri* occurs are not more similar to the native range environments than random; although for CliMond the result was near significance (p = 0.064 and p = 0.196; CliMond and PRISM respectively). Despite a niche shift, there remains considerable overlap between the native and invaded range niches (*D* = 0.32 and 0.28 for CliMond and PRISM respectively; Figs 1, S2, S3).

### SDMs generated from all occurrences in the native and invaded range

To generate SDMs, we obtained records from publicly available sources (GBIF and EDDMaps) and land manager records (see Methods for details) and filtered records to remove duplicates or errors. Within the filtered dataset, occurrence points were unevenly distributed because of variation in natural occurrences and sampling biases. We downsampled the dataset to a 50 km grid to minimize the disparity in sampling density among geographic areas (hereafter: native + invaded dataset). We explored SDMs built using Maxent and Boosted Regression Trees (BRT: see the electronic supplementary materials for BRT results).

**Figure 2:**
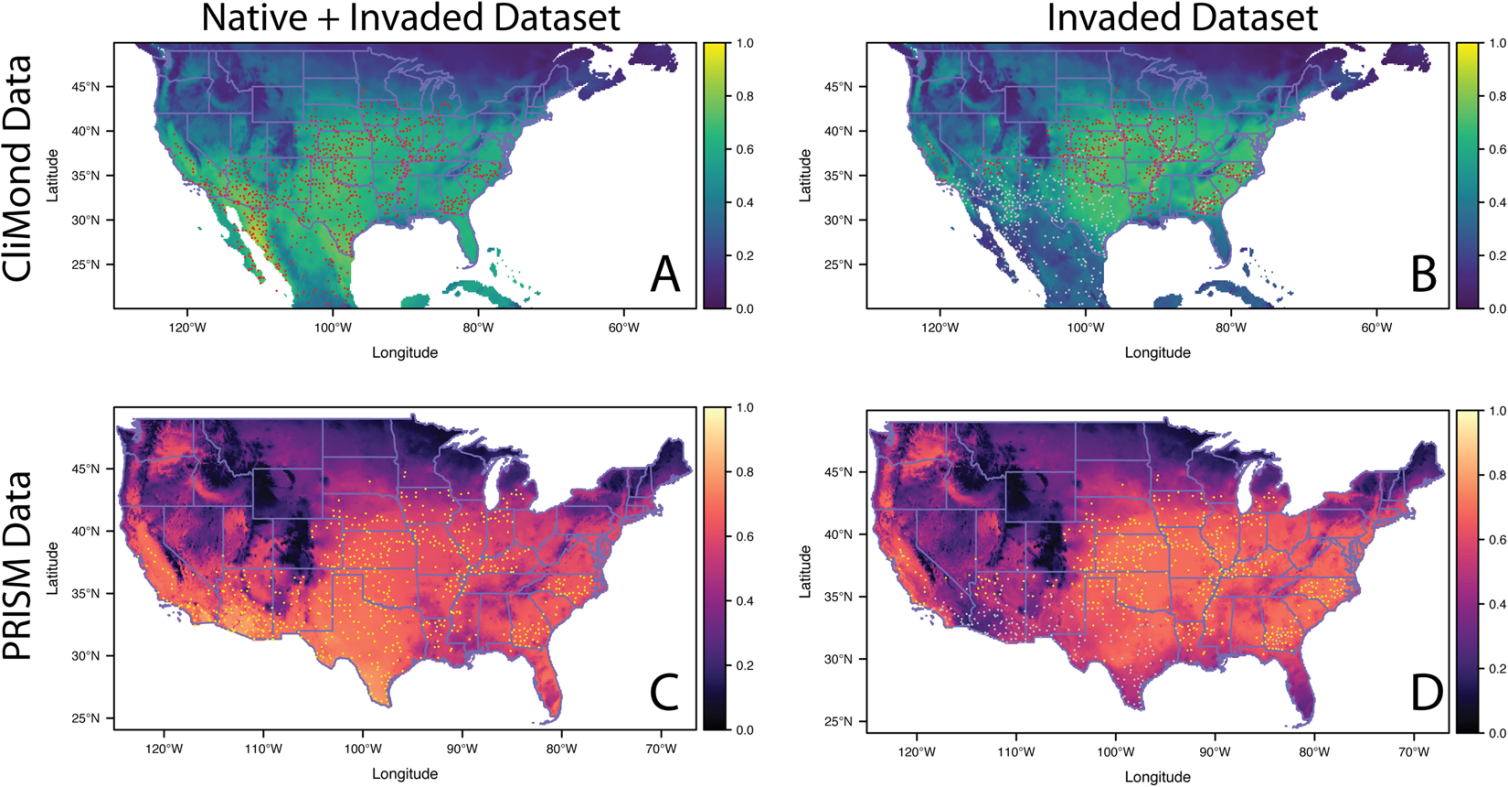
SDM model projections of *Amaranthus palmeri* generated using CliMond and PRISM variables for the native + invaded dataset and invasive only dataset. All projections are means of 25 model runs. Panels A and B were generated using CliMond environmental variables (blue/green color palette) and panels C and D were generated using PRISM variables (pink/purple color palette). Panels A and C used the native + invaded dataset for model building and panels B and D used the invasive only dataset. In panels A and B the occurrences included in the model are shown in red and in panels C and D the occurrences included in the model are shown in yellow. All other occurrences are in gray. The complementary log-log predicted probability for each raster cell is indicated by the color corresponding the bars to the right of the panels.

Species distribution models (SDMs) built with the native + invaded dataset indicated that a large portion of the conterminous United States and northern Mexico have high predicted probabilities of occurrence. For both the CliMond (projections in blue/green color palette) and the PRISM (projections shown in pink/purple color palette) models, areas of highest probability of occurrence included large portions of the desert Southwest, the Central Valley of California, most of the Midwest, the Southeast, the Mid-Atlantic, and southern New England (Fig. 2A,C). Areas predicted to have low probability of occurrence included mountainous regions and the highest northern latitudes of the United States (Fig. 2A,C). The SDMs based on PRISM data, which are centered on 1995 (compared to CliMond data, which is centered on 1975), showed higher predicted probabilities of occurrence at higher latitudes, with urban areas in the northern parts of the range having slightly higher probabilities of occurrence than surrounding rural areas (Fig. 2C).

Both CliMond and PRISM models had high predicted probabilities in a number of areas that are currently either unoccupied (e.g. the Palouse) or potentially unreported (e.g. Alabama, the Mid-Atlantic region, Ohio). Many of these areas report the presence of the species in the state but do not provide georeferenced occurrences and thus were not used to train our models (e.g. Maryland, Pennsylvania, New York; Fig. 2A,C). By contrast, the models predicted that recently colonized areas at the northern range margin (e.g. Minnesota, Wisconsin, Michigan) had marginal habitat suitability.

Both CliMond and PRISM models had low discrimination (AUC scores: 0.56–0.62), but moderately high model accuracy (TSS scores: 0.69–0.70; Table S1). Models performed similarly or marginally better in the native range than invaded range by most metrics (CliMond: Native-AUC: 0.67 v. Invaded-AUC: 0.61, Native-TSS: 0.53 v. Invaded-TSS: 0.49; PRISM: Native-AUC: 0.63 v. Invaded-AUC: 0.63, Native-TSS: 0.23 v. Invaded-TSS: 0.51).

**Figure 3:**
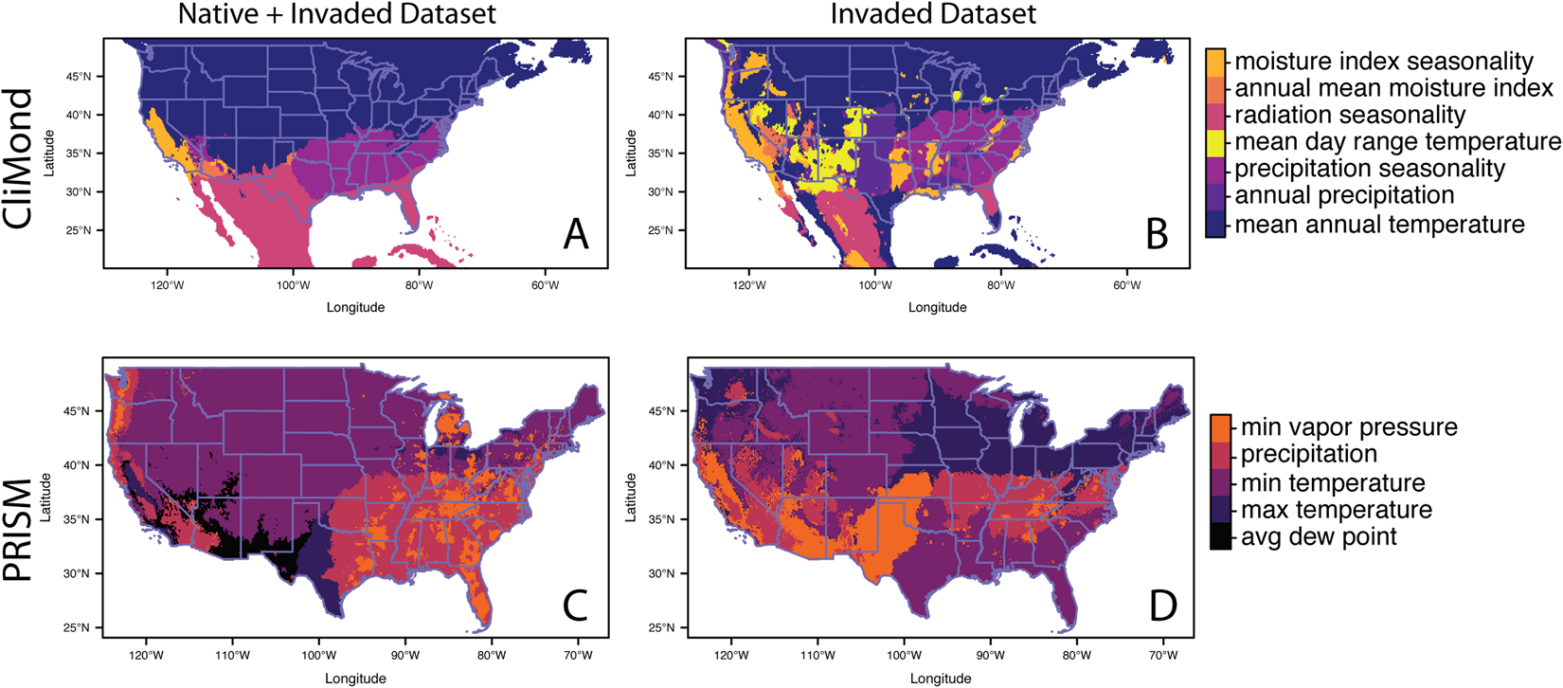
Limiting environmental factors on predicted probability for *A. palmeri*. The map depicts which environmental factor in the Maxent models contributes most negatively to the predicted probability of occurrence in each raster cell. The panels mirror the maps in the previous figure. Panels A and B are CliMond variables, panels C and D are PRISM variables, panels A and C are for models built with the native + invaded dataset and panels B and D are for models built with the invasive only dataset. Maps show are representative of the limiting factors by geographic region for the 25 models in the results.

For CliMond models, average annual temperature and radiation seasonality had the greatest variable contributions, 47.4% and 24.4% respectively (Table 1). For PRISM models, average minimum and maximum temperatures had the greatest variable contributions, 34% and 33% respectively (Table 1). The probability of occurrence along the northern range margin and in the Mountain West was most limited by low temperature (mean annual temperature for CliMond and low mean minimum temperature for PRISM; Fig. 3A,C). The probability of occurrence in the Southeast was most limited by high precipitation (CliMond and PRISM) and high vapor pressure (PRISM).

**Table 1:** Mean and standard errors of relative contributions of environmental variables included in Maxent models. Models were built using climate variables from CliMond or PRISM.

Variable	Bioclim #	Native + invaded Dataset				Invaded Dataset	
		Climate Only		Climate + Land Cover		Climate Only	
		Mean	SE	Mean	SE	Mean	SE
**CliMond Environmental Variables**							
Mean annual temperature	Bio 1	47.38	2.95	18.97	1.79	40.19	2.62
Mean diurnal temperature range	Bio 2	3.45	0.6	0.92	0.22	8.98	1.24
Annual precipitation	Bio 12	3.52	0.66	2.14	0.43	19.92	3.23
Precipitation seasonality	Bio 15	4.16	0.75	3.00	0.37	2.19	0.48
Radiation seasonality	Bio 23	24.46	2.17	7.91	0.97	11.11	2.00
Annual mean moisture index	Bio28	10.32	1.49	5.74	0.81	5.56	1.14
Moisture index seasonality	Bio31	6.71	1.36	1.72	0.4	12.04	1.62
Land Cover	—	—	—	59.6	1.54	—	—
**PRISM Environmental Variables**							
Maximum temperature	—	33.37	4.05	16.17	1.89	14.34	3.49
Minimum temperature	—	34.72	3.95	15.31	2.16	36.25	4.83
Precipitation	—	6.93	1.10	2.66	0.44	10.89	1.87
Maximum vapor pressure deficiency	—	6.00	1.35	3.45	0.83	2.99	0.85
Minimum vapor pressure deficiency	—	13.5	1.58	4.60	0.84	11.13	1.71
Dew Point	—	5.48	1.36	1.13	0.64	24.4	4.77
Land Cover	—	—	—	56.69	1.48	—	—

Models were built with occurrences from either the native + invaded dataset or the invaded dataset. For the native + invaded dataset, models included either climate variables only or climate variables plus land cover. All metrics are based on the averages of 25 model runs (see methods).

Including land cover in the models modestly increased model performance as measured by AUC (0.63–0.66) but not TSS (0.60–0.61; Table S1) and increased the predicted probability of occurrence in urban centers, particularly in northern regions (Electronic supplementary material, Fig. S4). Land cover also had a high relative variable contribution (60% for CliMond models, 57% for PRISM models; Table 1).

### SDMs generated only from occurrences in the invaded range

It is not clear if SDMs of invasive species perform better when generated from the entire range or the invaded range^6,15,16,19^, therefore we also built SDMs with occurrence records from only the invaded range. Similar to models built with native + invaded occurrences, these models predicted high suitability in most of the invaded range (i.e. central and southeastern United States); however, they performed comparatively worse in predicting occurrences in the native range (Fig. 2B,D). By contrast, the invaded range models were more successful in predicting occurrences at the northern range margin (Fig. 2B,D).

Similar to models based on data from the native + invaded range, models based on only the invaded range (both CliMond and PRISM) had low discrimination ability (AUC: 0.60–0.64; Table S1) and moderate accuracy (TSS: 0.64–0.68; Table S1). The most important environmental variables were mean annual temperature (40%) and mean annual precipitation (20%) for CliMond, and mean minimum temperature (36%) and dew point (24%) for PRISM (Table 1). The highest contributing variables for both climate datasets were the same as for the native + invaded model.

As with the native + invaded range model, low temperature was limiting for habitat suitability at the northern range margin. However, the invaded range models also indicated that a broad temperature range limited suitability at the northern range limit (Fig. 3B,D).

### Range expansion dynamics

**Figure 4:**
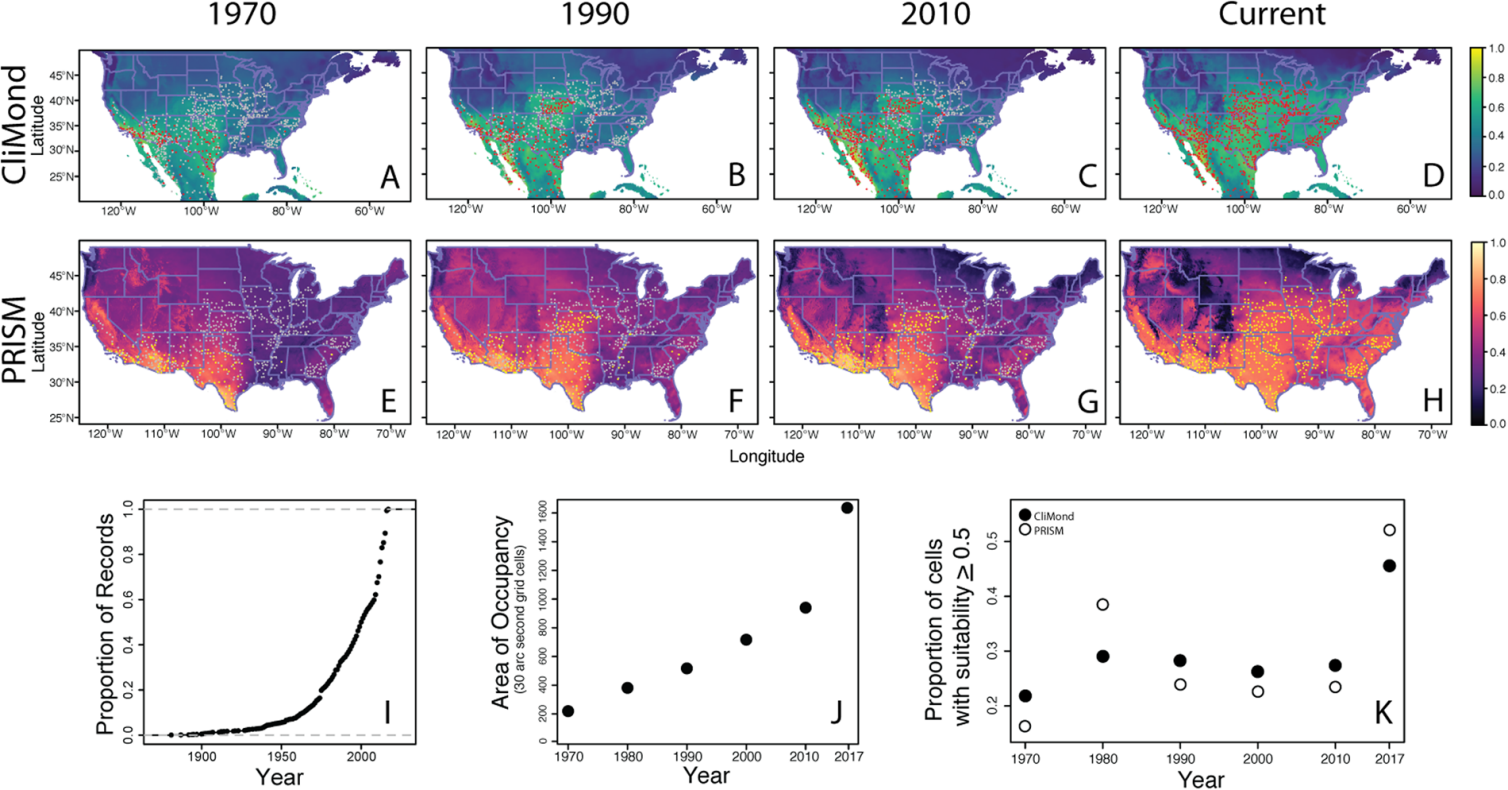
Sequential historical models and time series analyses of *A. palmeri*. Panels depict models built with occurrence records that include data up to a given year (1970, 1990, 2010). Projections on the top row (panels A–D) were built with CliMond environmental variables and those on the second row (panels E–H) were built with PRISM environmental variables. Occurrence records used to build models are in red (A–D; CliMond) or yellow (E–H; PRISM) all other occurrences are in gray. The complementary log-log predicted probability for each raster cell is indicated by the color corresponding the bars to the right of the panels. Metrics of range expansion are on the bottom row. The panel I depicts the accumulation of records through time. The panel J depicts the change in Area of Occupancy (measured in number of 30 arcsecond grid cells) over time. Lastly, the panel K depicts the average predicted probability over all grid cells for models built at each time period.

The number of occurrence records and geographic range increased dramatically after 1950 (95% of records since 1950), when *A. palmeri* was first reported to spread outside of its native range and >50% of records are from the last 10 years (Fig. 4I).

An increasing pace of invasion was also evident in the change in area of occupancy through time (area of occupied grid cells; Fig. 4J). The relationship was linear until approximately 2000–2010 (linear AICc: 76.1; log-linear AICc: 76.7); however, since 2010, an accelerating (log-linear) relationship provided a better fit (linear AICc: 97.2; log-linear AICc: 89.8).

Models built using data from 2010 and before did not forecast the extent of the current invaded range (Fig. 4A–H; model evaluations in Table S2). In the modelled range, sequential historical models had low overall discrimination (AUC: 0.58–0.63) and moderate accuracy (TSS: 0.62–0.74). Additionally, historical models were not able to predict future occurrences better than random, even when evaluations were limited to regions with only analogous climates (AUC: 0.53–0.55, TSS: −0.12–0.16). When projecting habitat suitability across North America, the proportion of suitable habitat only increased modestly from ~20% (1970) to 25–30% (1980 to 2010), then abruptly increased to ~50% in 2017 (Fig. 4K).

Occurrences in the native range were spatially autocorrelated at all three spatial scales assessed (1 and 10 nearest neighbors, all neighbors within 10 km: Join-count pseudo *P* < 0.0001). By contrast, in the invaded range, occurrences were spatially autocorrelated only at the larger spatial scales (*P* < 0.0001 for both 10 nearest neighbors and all neighbors within 10 km, *P* = 0.74 for single nearest neighbor). The weaker autocorrelation at the finest spatial scale suggests that stochastic long-distance dispersal events likely contributed to the invasion process rather than wave-like spread from nearest neighbors, alone.

### Projected future distributions

**Figure 5:**
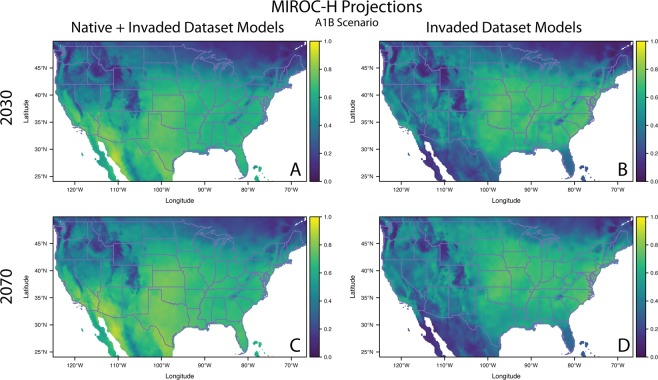
Model projection of *A. palmeri* under future climate change scenarios. The projections are for the MIROC-H Global Circulation Model under the A1B emissions scenario. Panels A and B are based on projected environmental variables in the year 2030 and panels C and D for year 2070. The panels A and C are projections based on models built with the native + invaded dataset and panels B and D are based on models built with the invasive only dataset. The complementary log-log predicted probability for each raster cell is indicated by the color corresponding the bars to the right of the panels.

Under all future climates, using the native + invaded range models, the area of high habitat suitability expanded and models projected greater habitat suitability in the Upper Midwest (MI, WI, MN, ND, SD), Pennsylvania and New York. Projections of the invaded range models predicted a similar northern expansion of suitable habitat, albeit accelerated and more extensive. For example, regions of high suitability are evident in central Minnesota and North Dakota by 2030, and by 2070 the majority of both states are deemed highly suitable (Figs 5A–D, S5–S7).

## Discussion

Species distribution models based on current records of occurrence accurately predicted *A. palmeri’s* invaded range and indicated that temperature plays a particularly important role in limiting habitat suitability at the northern range margin. SDMs performed better when we used occurrences only from the invaded range, rather than including the native range as well. Under future climate scenarios, our models predicted northward range expansion and increased suitability in regions that are already occupied but of moderate suitability (e.g. upper Midwest). Unfortunately, SDMs performed quite poorly when we attempted to predict the current invaded range from earlier phases of the invasion history (e.g. occurrences until 1970, 1980, 1990, 2000, 2010). Analyses of invasion dynamics suggest that stochastic movements across the landscape have contributed importantly to rapid range expansion, rather than a wave-like spread, alone. Our models make robust predictions about incremental expansion in the future but suggest caution when employing SDMs to predict the potential geographic range of invasive species that are far from equilibrium with the environment.

We used a diverse set of modelling approaches and all of them had modest discrimination ability. This was evident by low AUC scores (but TSS indicated moderate to high accuracy), weak signal in individual environmental variables, and predictions of low habitat suitability in currently occupied regions at the range margin. Discrimination ability and predictions of habitat suitability were consistent across all versions of our models, suggesting that the modelling approach was not the likely cause. Instead, the climate datasets along with the biology and invasion history of *A. palmeri*, may have contributed to limited SDM discrimination. First, the climate datasets used (CliMond and PRISM) are constructed based on mean data over a 30-year timeframe (CliMond: 1961–1990; PRISM: 1970–2010). Therefore, it is possible that our models are conservative with respect to predictions of habitat suitability, and occurrences along the range margin, particularly in the north, may reflect recent warmer than average years. Second, it is possible that climate has not been the main driver of distributional limits but rather that they have been caused by other factors (e.g. dispersal or biotic interactions). For example, range expansion may have been limited by the availability of disturbed, low-competition habitats^35,36^ that have become more common in the past 70+ years with industrialized agriculture^37^. Third, annual plants such as *A. palmeri* can often exploit a broad range of climates by exploiting only the fraction of the year that is suitable via altered timing of germination and reproduction (i.e. niche construction^38^). Last, populations may have adapted to novel environments during range expansion, which has resulted in a broad set of suitable climates across the species’ range and a weakened signal of individual environmental variables in SDM construction. Together, these historical and biological factors may contribute to modest model discrimination/accuracy but also provide important insight for future work on the causes of distributional limits. We consider each of these issues in detail below.

*Amaranthus palmeri* remained within the bounds of its native range until the middle of the twentieth century but quickly began to spread by the 1970s and 1980s^28^. We took advantage of the detailed time series of occurrence data to ask how SDMs performed at sequential stages of the invasion process. Models built with occurrence records until 1970, which were primarily from the native range and early invasion, predicted very little of the extent of the eventual invaded range. Models built using occurrence records until 1980, 1990, and 2000 also performed poorly in forecasting the eventual invaded range. Qualitative inspection of projections indicate that models were successful in predicting the invasion of geographic regions immediately adjacent to the occupied range (i.e. short periods into the future) but failed to predict over broader geographic areas (and longer time scales). These results imply that climate was not an important factor in the early stages of the invasion and that dispersal limitation contributed to limits on range expansion. Our models that incorporated land cover made similar predictions about the extent of invasion, but also emphasized that human disturbance was likely an important factor in range expansion. Similarly, a meta-analysis by Simberloff *et al*.^24^ found that most invasions of native plants are not limited by climatic variables and instead are associated with anthropogenic change, such as alteration of grazing and fire regimes. The association of *A. palmeri* with natural and human-caused disturbed habitats may in part explain the original restriction and current movement of the species.

Our results also suggested that stochastic long-distance dispersal contributed to rapid range expansion throughout the invasion process. The total area of occupancy for *A. palmeri* in North America increased linearly during most of the invasion history but began to accelerate rapidly approximately in the last decade (2010–2017). We also found that occurrences in the invaded range were not spatially-autocorrelated at fine spatial scales (nearest neighbors) indicating that stochastic movement (e.g. long-distance dispersal events) has likely been important. This result is consistent with observations from other systems, and theoretical models, which have suggested that rapidly expanding ranges often spread via short-distance dispersal at range margins coupled with rare long-distance stochastic dispersal^39,40^. Such rapid and stochastic invasion is most likely to occur when an invader is far from equilibrium with the environment (e.g. climate), with large amounts of suitable but unoccupied territory.

Although we detected a recent acceleration in invasion speed and stochastic spread, our models suggest that *A. palmeri* is closer to reaching the boundaries of its potential range. Models built with the most recent datasets (2017) best predicted large geographic areas that have recently been invaded but do not have occurrences included in model building (e.g. Maryland, Pennsylvania, New York, and portions of the Southeast and Upper Midwest; USDA Plant Database; https://plants.usda.gov); whereas, models built with older datasets underpredicted areas at risk for future invasion. Similar to our results, Václavík and Meentemeyer^41^ found that early in the invasion history of *Phytophthora ramorum*, models underpredicted areas at risk for future invasions, but that late in the invasion more models readily predicted unoccupied areas that were later invaded. Therefore, our models based on current datasets may likely make more robust predictions about future invasion than those based on data from earlier in the invasion process.

One of the difficult decisions faced by modellers of invasive species is whether and how to use occurrence information from the native range in modelling and projecting invasive ranges^6^. In many cases, using native plus invasive records overpredicts habitat suitability in unoccupied areas^12^; whereas, using only the invaded range better predicts potentially suitable unoccupied areas. In our models, we found that using occurrences from the native + invaded range underpredicted some areas that have already been invaded (i.e. the Midwest, Southeast, and Mid-Atlantic; Figs 2A,C, S8). These newly-invaded regions had higher predicted habitat suitability when models were built using data from only the invaded range. Therefore, like many other invasive species, newly-invaded areas are best predicted by models built only with invaded occurrences^6,19^. This is likely because the native range is a narrow subset of climates compared to the invaded range. Indeed, our niche analyses showed that the native and invaded ranges occupied different, but overlapping, climatic niche space, which is common for many invasive species^19,42^. This niche shift makes it potentially problematic to model invaded regions using native-only models or models where a high density of native occurrences might heavily bias the SDM.

Range expansion may have been facilitated by adaptation and/or admixture. *Amaranthus palmeri* may have adapted to novel environments (e.g. temperature and precipitation regimes) during northern and eastern range expansion and thus differentiated from native genotypes. Thus far, there is evidence that resistance to glyphosate application quickly evolved^43^, which has greatly enabled its spread into agricultural fields^28^. Because SDMs assume that all populations have the same climate niche, local adaptation across the range violates this assumption and may produce biologically unrealistic predictions^7–9^. This may make it more important to build detailed, regionally-informed models to capture patterns of relevant environments and genotypes. In addition to adaptation from standing genetic variation, admixture from closely-related species could also contribute to range expansion and confuse SDMs. Admixture has been documented with *A. tuberculatus*^44,45^, a widespread species in the central United States (ranging from Canada to Mexico) but it’s importance for adaptation to northern and eastern environments remains unclear. Nevertheless, it is possible that *A. tuberculatus* has served as an important source of adaptive genetic variation during range expansion. Given the potential for evolution to influence invasion dynamics in this system, it would be particularly profitable to build SDMs that incorporate information on functional traits and adaptive differentiation^46,47^.

## Methods

### Study system

*Amaranthus palmeri* is a dioecious, wind-pollinated, obligate outcrossing, annual plant that harbors substantial genetic diversity within and among populations^26,28,31,45^. In its native range, *A. palmeri* occurs in dry, itinerant stream and riverbeds and other habitats subject to frequent disturbance^25,26,28^. *A. palmeri* has high photosynthetic rates, grows rapidly, and can quickly deplete soils of nutrients^48–50^. Its seeds can germinate throughout the growing season and plants can successfully reproduce at almost any size or age^26,28^. This combination of traits has predisposed the species for success in agricultural fields and other disturbed areas^26^. There is also evidence for widespread herbicide resistance (e.g. glyphosate) and other adaptations to weed control, including delayed germination and rapid development^28^.

### Species occurrence records

#### Data sources

We gathered 3,967 occurrence records, ranging in dates from 1896–2017, from open-access databases and herbarium networks including: Global Biodiversity Information Facility (GBIF; www.gbif.org; Appendix A), Early Detection Distribution Mapping System (EDDMapS; www.eddmaps.org; Appendix A), and the Southwest Environmental Information Network (SEINET; swbiodiversity.org/seinet; Appendix A). We also incorporated 434 county-level occurrence records (approximately the same geographic precision as the minimum for the primary environmental dataset; see below) for several Midwestern and Southeastern states (AK, IA, IL, IN, MI, MO, MN, MS, NE, OH, SD, TN; Appendix A) where few detailed records were available. These data were provided directly by state land managers; exact localities were not provided to protect landowner identity. For these county-level data we used the geometric centroid of each county as the location of occurrence.

### Environmental data

To generate SDMs and quantify niche dynamics, we used climate data extracted from CliMond (www.climond.org)^51^ and PRISM (prism.oregonstate.edu). From CliMond, which is based primarily on climatological data from 1961–1990, we extracted data for all 35 bioclimatic variables at 10 arc-minute resolution (the finest scale available for all GCMs used). From these 35 variables, we identified seven predictor variables: mean annual temperature, mean diurnal temperature range, annual precipitation, precipitation seasonality, radiation seasonality, mean annual moisture index, and moisture index seasonality. We selected these variables because they were not strongly correlated (Pearson r < 0.8) and described the major axes of climatic variation along the first two axes of a PCA of the current range. We also extracted seven climate variables, including one composite variable, from PRISM, which is based on data collected from 1981 to 2010 at 30 arc-second resolution. For PRISM, we retained all variables except mean annual temperature, which was highly correlated with minimum and maximum temperature. We retained more variables than is typical for many SDMs to provide more robust predictions of habitat suitability in unoccupied regions and under future climate projections^14^. For a subset of SDMs, we included the land cover layer as an additional categorical variable. We obtained data on land cover (2011 land cover characterizations, www.mrlc.gov/nlcd11_data.php) from the Multi-Resolution Land Characteristics Consortium (www.mrlc.gov/) and rasterized the image to an 800 m resolution using the R ‘raster’ package^52,53^.

### Tests of niche differentiation

We tested for niche differentiation between the native and invaded range following Broennimann *et al*.^34^ using the ‘ecospat’ package in R^54^. Using climate data from CliMond and PRISM, we calculated the extent of the native and invaded niche (niche breadth) and the native and invaded niche centroid, which was weighted by the number of occurrences and the availability of the environment. We defined total niche space using principal components analysis (PCA-env^34^) for the 100 km background buffers used for building SDMs (i.e. see ‘SDMs for the native + invaded range and invaded range only’ section below). We quantified niche overlap using Schoener’s *D*, which varies between 0 and 1 (zero and complete niche overlap, respectively). We tested if the invaded niche was more similar to the native niche than expected by chance using a permutation test (N = 999 permutations) for niche similarity (i.e. are invaded occurrences in the invaded range more similar to the native niche than expected by chance)^34^. We also characterized the niche breadth and overlap along individual environmental axes (Electronic Supplementary Materials).

### Data preparation for SDMs

We manipulated and analysed spatial data in the R environment version 3.4.1^52^ using the geospatial data abstraction library (GDAL) implemented in package ‘rgdal’ and the Geometry Engine Open Source (GEOS) implemented in the package ‘rgeos’^55^. To manipulate spatial point data, we used the ‘sp’ package^55–57^.

Prior to analyses, we removed all records that were geographic outliers, duplications, or had very low coordinate precision (<0.1 decimal degrees, ~10 km precision), which left 1,453 records (hereafter: filtered dataset). Within this filtered dataset, occurrence points were highly unevenly distributed because of variation in natural occurrences and sampling biases. In particular, the species’ native range (SW U.S., NW Mexico) was far more heavily sampled than nearly all of the invaded range. To minimize the disparity in sampling density among geographic areas we down-sampled occurrence records to a 50 km grid, resulting in dataset containing 791 occurrences (hereafter: native + invaded dataset). The SDMs we present in this paper were generated using this native + invaded dataset but for comparison, we also performed analyses on the 1,453-record filtered dataset (reported in Electronic supplementary material, Fig. S8).

### SDMs for the native + invaded range and invaded range only

We built SDMs using Maxent version 3.4.1^58,59^ and Boosted Regression Trees (BRT)^60^. The two approaches produced similar results and we therefore focus on the Maxent models and present BRT methods and results in the Electronic Supplementary Materials. One set of models was built based on the native + invaded occurrences (n = 791) and a second set included only those in the invaded range (n = 427). The invaded range was defined using expert-drawn range maps from before most of the invasion occurred^25^. We used the ‘dismo’ package^61^ to build Maxent models and the ‘gbm’ package^62^ to build boosted regression tree models. We also used the packages ‘ROCR’^63^ and ‘rmaxent’^64^ for evaluating models and for generating rapid model projections. We projected models using the ‘project’ function in the package ‘rmaxent’^64^. Last, we visualized all projections using the package ‘rasterVis’^65^.

For Maxent, we generated background points by selecting points from a polygon object generated by drawing 100-kilometer buffers centered on each presence point and dissolving overlaps. We randomly selected 10,000 points from the polygon while accounting for differences in area of raster cells at higher latitudes^10^. Background points generated this way capture the relevant climatic variation within the area of dispersal^41,66^, while balancing the tendency for inflated validation metrics (e.g. AUC) resulting from sampling large areas^67^.

We trained the model with 80% of the occurrence and background data and withheld 20% for model validation. Models were built allowing for all feature types^10^ and with a regularization parameter of 3 (‘betamultiplier’), which had the best performance among models where we varied the regularization parameter from one to five^68^. Finally, we built five models with five-fold cross validation (25 total models) to account for potential variation in model parameters.

We used Multivariate Environmental Similarity Surfaces (MESS) to classify analogous climate regions in North America that were used for two purposes: (1) to identify appropriate unoccupied habitats (e.g. areas under threat of invasion) for model projections and (2) to calculate evaluation metrics for sequential historical models using only relevant future occurrence records (see ‘Range expansion dynamics’ below). For all models, we calculated MESS based on background points within the 100 km background buffers used for model-building^7,9^. Analogous climate areas were defined as having similarity values greater than 0. For models built with the native + invaded and invaded range datasets, we found that the conterminous United States was deemed as climatically similar enough to be suitable for accurate model projection.

To determine which climatic variables most negatively influenced the probability of occurrence, we generated limiting factor maps using the ‘limiting’ function in the package ‘rmaxent’^64^. This function identifies which bioclimatic variables weigh most negatively on the predicted probability value at each raster cell in a Maxent projection^9,64^. Identifying limiting factors in unoccupied regions can indicate which factors most likely hinder range expansion.

#### Model evaluation

We evaluated model performance using two statistics: Area Under the Curve (AUC) and True Skill Statistic (TSS). AUC (also referred to as ‘ROC’) scores evaluate how well a model performs relative to random chance and gauges the model’s discrimination ability^69^. We computed two values for AUC: (1) AUC-train, calculated using training data to determine how well the model predicts the data used in model building and (2) AUC-test, calculated using testing data retained for validation to evaluate the ability of the model to predict new information.

We calculated the True Skill Statistic (TSS)^70^, which describes the ability of a model to correctly classify presences and background data.

$${\rm{TSS}}={\rm{True}}\,{\rm{Positive}}\,{\rm{Rate}}\,({\rm{TPR}})+{\rm{True}}\,{\rm{Negative}}\,{\rm{Rate}}\,({\rm{TNR}})-{\rm{1}}$$

TSS values range from 1, which is indicative of perfect accuracy (i.e. models predict all presences), to −1, which is indicative of perfect inaccuracy (i.e. models do not correctly predict any presences). TSS = 0 indicates that the model performs no better than random. We evaluated TSS at a model-dependent threshold value, at which the sum of TPR and TNR was maximized (threshold values ranged between 0.5 to 0.65)^71,72^.

### Range expansion dynamics

To examine the temporal dynamics of invasion we conducted two series of analyses on five data subsets each of which contained all of the records in the native + invaded dataset that had been collected up to the years 1970, 1980, 1990, 2000, and 2010 (n = 146, 235, 309, 390, 458); prior to 1970, there were too few records to build reliable models. First, to characterize the fine-scale rate of spread across North America, we calculated the Area of Occupancy (AOO) as the number of grid cells (30 arcsecond size) with records. We also built SDMs at each time point (same methods as for the datasets described above) to visually compare projections and to quantify the proportion of grid cells with a predicted probability of occurrence ≥0.5. We evaluated these models in two ways. First, we calculated evaluation metrics using a testing dataset withheld during model building. Second, we evaluated the models’ ability to predict future invasion by calculating evaluation metrics (AUC and TSS) using all future occurrence records found in analogous climate space. For sequential historical models, only subsets of the US contained analogous climates. Therefore, only occurrence records that occurred within those regions were used to calculate evaluation metrics base on future prediction of occurrences.

Second, we examined whether the pattern of range expansion was more consistent with wave-like versus stochastic movement. Wave-like movement causes higher levels of spatial autocorrelation than stochastic movement among occurrences because the bulk of propagule movement is local^73^. We used the filtered dataset (2017) to assess spatial autocorrelation at three spatial scales: single nearest neighbor, ten nearest neighbors, and all neighbors within a 50 km radius. To determine the extent of spatial autocorrelation, we calculated the join-count statistic which tallies the number of links between nearest neighbors (i.e. presence-presence, presence-absence, absence-absence). To determine number of same-type joins that would be expected by chance we generated 999 permuted datasets and calculated the join-count statistic for each. To determine if patterns of spatial autocorrelation were different between native and invaded ranges, we performed analyses separately for each region. We used the ‘spdep’ package to perform spatial autocorrelation analysis^74,75^.

### Projected future distributions

To make predictions about potentially suitable future habitats, we obtained the same bioclimatic variables for CliMond from the general circulation models (GCM) CSIRO-Mk3.0 and MIROC-H for the A1B and A2 SRES climate scenarios (A1B is based on lower CO_2_, N_2_O, CH_4_, and SO_2_ emissions than A2) for 2030 and 2070 (IPPC IV SRES 2007^76^). The CSIRO-Mk3.0 and MIROC-H GCMs models provide predicted values for each of the CliMond bioclimatic variables and also perform well in the generation of future climate scenarios^51^. We projected models using the ‘project’ function in ‘rmaxent’^64^ for the native + invaded models and invaded only models and visualized the resulting shifts in distributions.

## Supplementary information

Electronic Supplementary Materials

## Data Availability

All data was gathered from publically available sources and will be made available in the format used for analysis at the Data Repository for the University of Minnesota (DRUM; www.lib.umn.edu/datamanagement/drum).
