# Supplementary material for: Species distribution models throughout the invasion history of Palmer amaranth predict regions at risk of future invasion and reveal challenges with modeling rapidly shifting geographic ranges
Source: Sci Rep. 2019 Feb 20;9:2426. doi: 10.1038/s41598-018-38054-9 (PMC6382853; doi:10.1038/s41598-018-38054-9)
Supplement: Supplementary file 1 — Electronic Supplementary Materials [file 41598_2018_38054_MOESM1_ESM.pdf]

Electronic Supplementary Materials

Species distribution models throughout the invasion history of Palmer amaranth predict regions at risk of future invasion and reveal challenges with modeling rapidly shifting geographic ranges

Authors: Ryan Briscoe Runquist, Thomas Lake, Peter Tiffin, and David Moeller

Table of Contents

1) Figure S1. PCA correlation plot of climate space for CliMond and PRISM 2
2) Figure S2. PRISM native versus invaded niche plot 3
3) Figure S3. Niche Differentiation along environmental axes 4
4) Table S1. Model Evaluation Metrics 5
5) Figure S4. SDM of full dataset with climate variables and land cover 6
6) Table S2. Model evaluations for models from different time periods................... 7
7) Figures of Future projections for alternate GCMs
    a. Figure S5. MIROC-H; A2 Future Projections 8
    b. Figure S6. CSIRO; A1B Future Projections 9
    c. Figure S7. CSIRO; A2 Future Projections 10
8) Figure S8. SDM of filtered Dataset 11
9) BRT Methods and Results 12
    a. Figure S8. BRT with tree complexity 2 17
    b. Figure S9. BRT with tree complexity 5 18
    c. Table S3. Variable contributions in BRT models....................................... 19
10) APPENDIX A: Occurrence Record Documentation 21

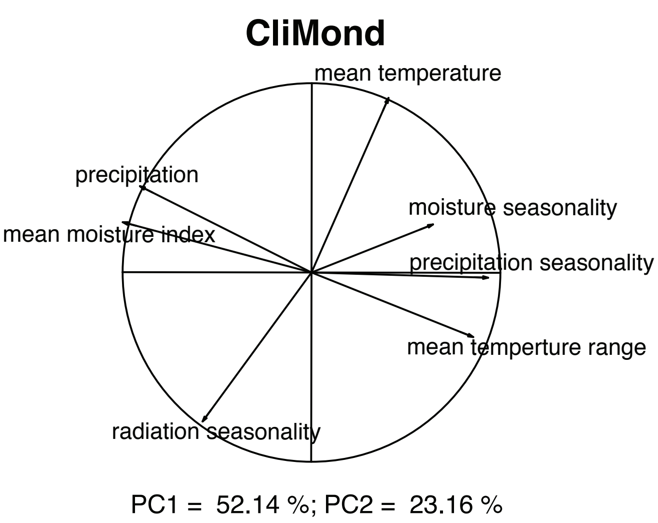

Figure S1. PCA correlation plot of climate space for CliMond and PRISM. (A) PCA of CliMond variables over the total environmental extent. The first two PCA axes accounted for 75.3% of the environmental variation; PC1 (52.14%) included mostly precipitation and moisture variables with high values indicating higher seasonality in moisture and precipitation and lower values indicating greater overall moisture and precipitation; temperature and radiation seasonality loaded most heavily on PC2 (23.16%), with high values indicating higher mean temperatures and lower values indicating greater seasonality in receipt of radiation. (B) PCA of PRISM variables over the total environmental extent. The first two PC axes accounted for 92.6% of the environmental variation; PC1 (49.76%) included temperature and vapor pressure variables, where lower values indicate higher temperatures (max and min) and higher vapor pressure deficiencies (max and min); Dew point, precipitation, and minimum temperature (to a lesser extent) all loaded on PC2 (42.82%) with lower values indicating higher precipitation, dew points, and minimum temperatures.

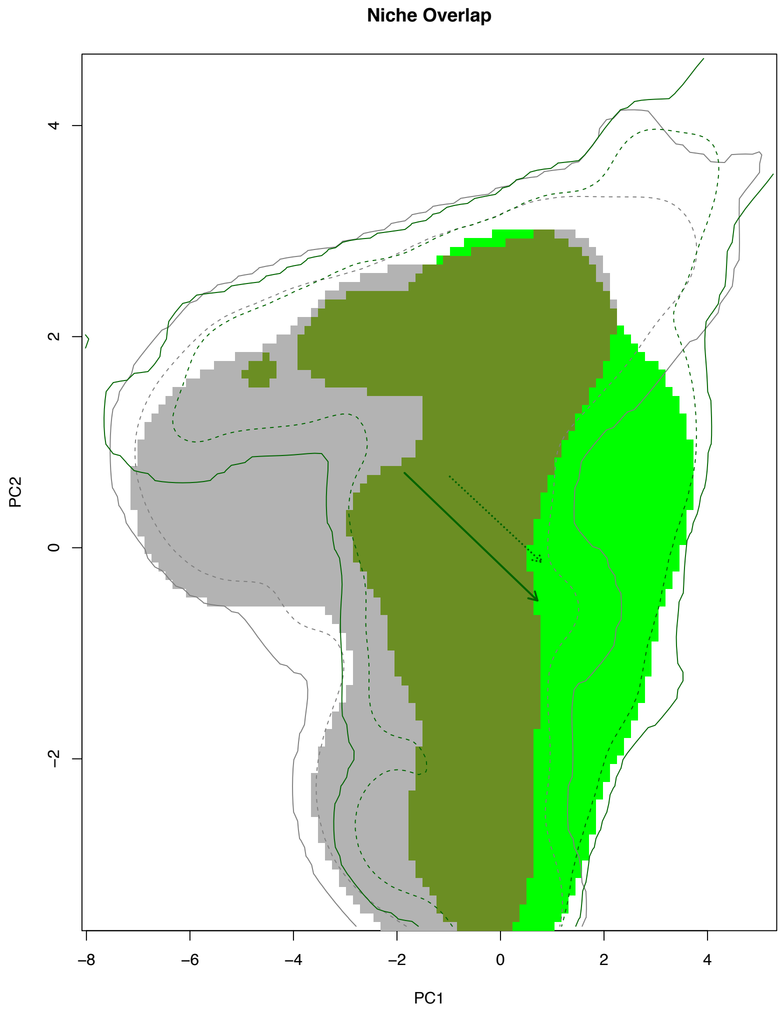

Figure S2. Niche breadth and shift of native versus invaded niche. Axes are principal components of PRISM variables included in the model. The total extent of the background environment in the native region is outlined in grey (solid = total niche space; dashed = 90% of extent). The total extent of the background environment in the invaded region is outlined in dark green (solid = total niche space; dashed = 90% of extent). The environmental space unique to the native niche is shown in gray (31.5% weighted loss of the original niche), the area of environmental space shared between the native and invaded niche is shown in olive-green (70% of the total weighted invaded niche), and the region of environmental space newly occupied by the invaded niche is shown in green (i.e. niche expansion; 30% if the total weighted invaded niche). The arrows represent the shift in the weighted centroid of occurrences from native to invaded niche (dark bold arrow), of the shift in the center of environmental space (lighter small arrow). The weighted niche overlap, calculated using Schoener's *D*, was 0.28.

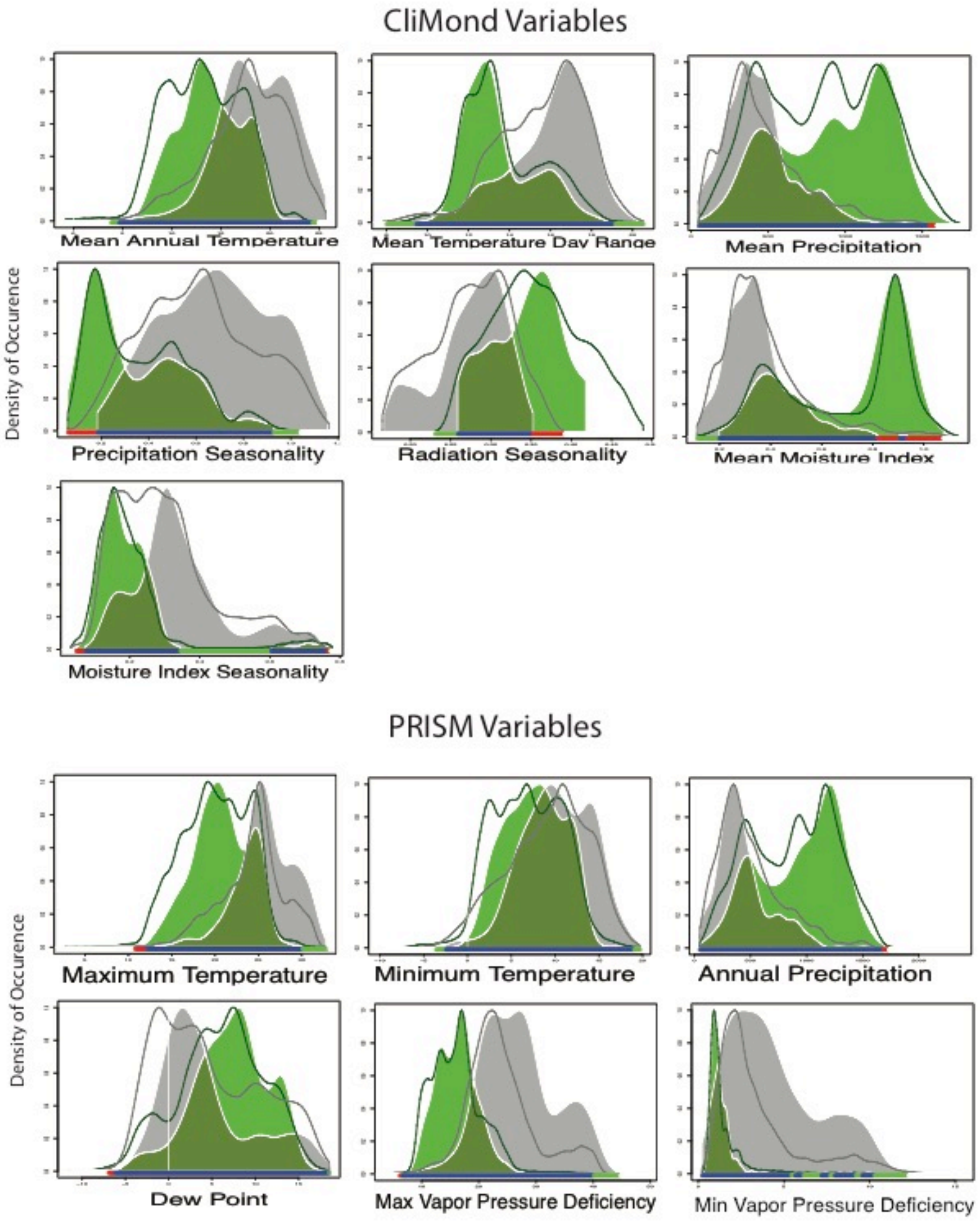

Figure S3. Niche Differentiation along environmental axes for CliMond and PRISM variables included in models. The environmental variables are along the x-axis and the density of occurrences or environments is along the y-axis. The density of the background environment in the native range is outlined in grey. The density of the background environment in invaded area is outlined in dark green. The environmental space of the native niche that is unfilled in the invaded area is shown in gray, the area of environmental space shared between the native and invaded niche is shown in olive-green, and the expanded region of environmental space occupied by the invaded niche is shown in green.

Table S1. Model Evaluation Metric means, standard errors, and ranges for the native + invaded dataset, native + invaded dataset including land cover, and the invaded range dataset for CliMond and PRISM climate variables. All metrics are based on the 25 Maxent models (see methods for further details).

	Native + Invaded Dataset					
	CliMond			PRISM		
	Mean	SE	Range	Mean	SE	Range
AUC-test	0.60	0.004	0.56-0.64	0.56	0.006	0.53-0.65
AUC-train	0.62	0.004	0.60-0.63	0.61	0.002	0.58-0.64
TSS	0.69	0.03	0.37-0.87	0.70	0.03	0.38-0.95
	Native + Invaded Dataset					
	CliMond + Land Cover			PRISM + Land Cover		
	Mean	SE	Range	Mean	SE	Range
AUC-test	0.63	0.004	0.60-0.66	0.63	0.005	0.57-0.67
AUC-train	0.65	0.002	0.63-0.68	0.66	0.005	0.63-0.67
TSS	0.60	0.02	0.35-0.77	0.61	0.03	0.38-0.82
	Invaded Dataset					
	CliMond			PRISM		
	Mean	SE	Range	Mean	SE	Range
AUC-test	0.60	0.006	0.57-0.63	0.61	0.006	0.56-0.69
AUC-train	0.64	0.004	0.61-0.67	0.63	0.003	0.61-0.66
TSS	0.64	0.04	0.36-0.93	0.68	0.03	0.36-0.89

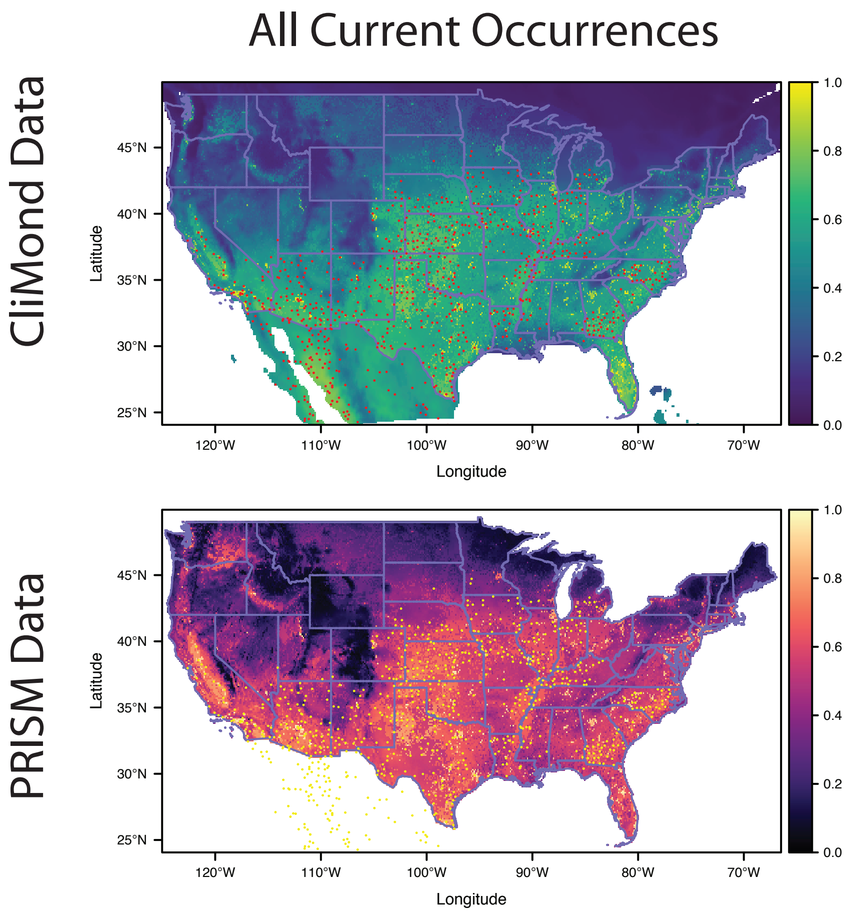

Figure S4. SDM model projections of *Amaranthus palmeri* generated using CliMond and PRISM variables plus land cover for the native + invaded dataset. All projections are means of 25 model runs. The top panel was generated using CliMond environmental variables and the bottom panel was generated using PRISM variables. In top panel the occurrences included in the model are shown in red and in the bottom panel the occurrences included in the model are shown in yellow. The complementary log-log predicted probability for each raster cell is indicated by the color corresponding the bars to the right of the panels.

Table S2. Model Evaluation Metrics for Maxent models based different time periods. Models were built using subsets of the native + invaded dataset with occurrence record dates up to the year specified.

	CliMond					PRISM				
	Model			Future Occurrence in analogous climates		Model			Future Occurrence in analogous climates	
	AUC-test	AUC-train	TSS	AUC	TSS	AUC-test	AUC-train	TSS	AUC	TSS
1970	0.60	0.65	0.63	0.54	0.16	0.62	0.65	0.69	0.53	-0.12
1980	0.59	0.64	0.66	0.53	0.05	0.58	0.63	0.71	0.52	0.02
1990	0.60	0.64	0.62	0.53	0.00	0.58	0.63	0.69	0.53	0.03
2000	0.61	0.65	0.63	0.55	0.02	0.62	0.64	0.74	0.55	0.07
2010	0.62	0.64	0.66	0.53	0.06	0.63	0.64	0.73	0.55	0.09

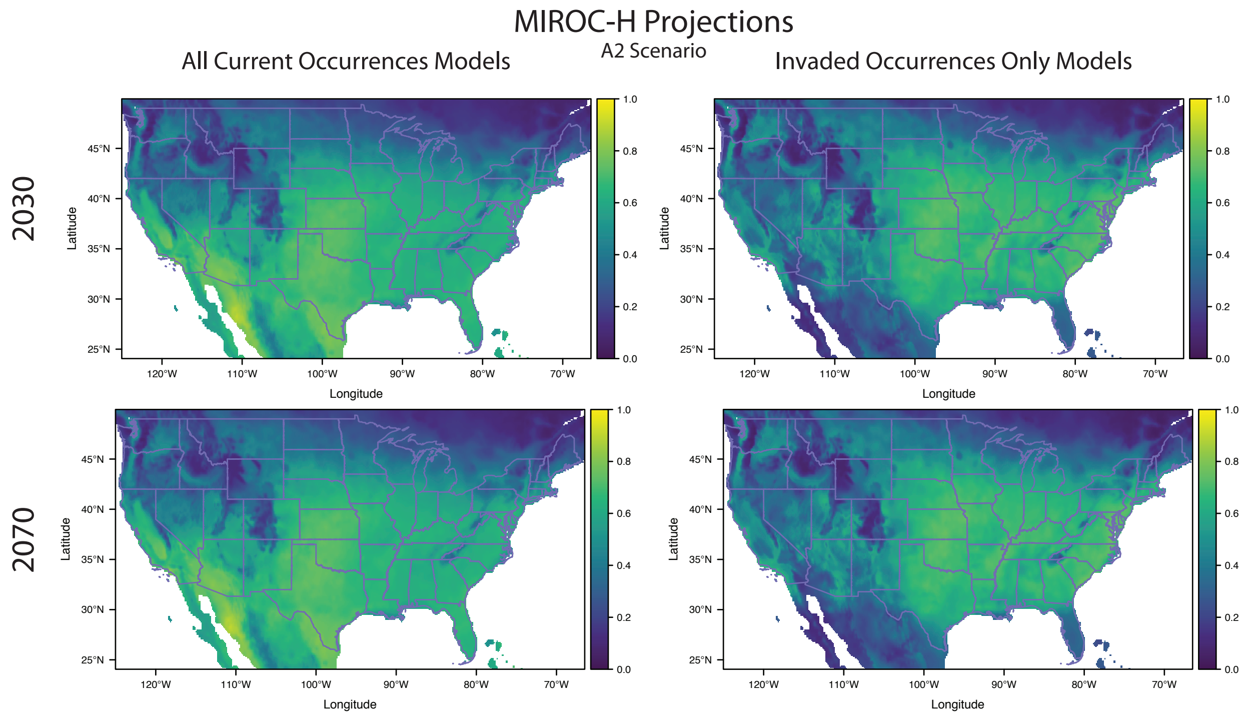

Figure S5. Model projection of *A. palmeri* under future climate change scenarios. The projections are for the MIROC-H General Circulation Model under the A2 emissions scenario. Panels on the top are based on projected environmental variables in the year 2030 and on the bottom for year 2070. The left panels are projections based on models built with the full dataset and panels on the right are based on models built with the invasive only dataset. The complementary log-log predicted probability for each raster cell is indicated by the color corresponding the bars to the right of the panels.

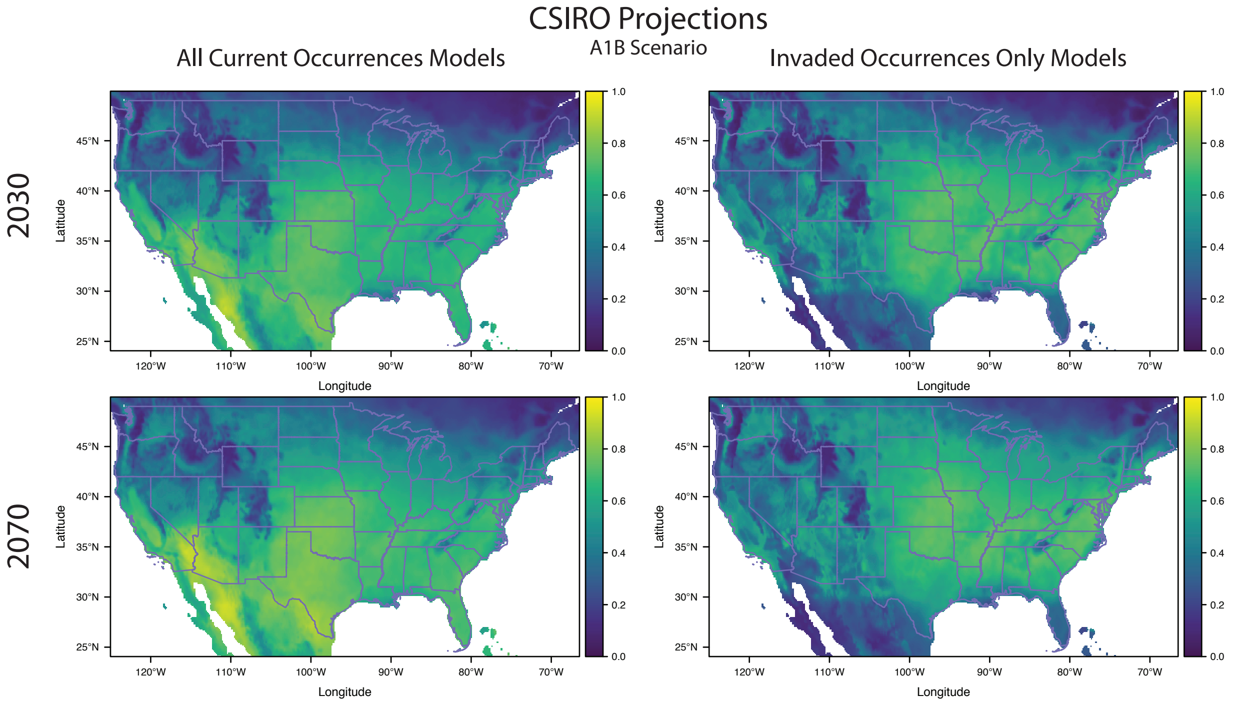

Figure S6. Model projection of *A. palmeri* under future climate change scenarios. The projections are for the CSIRO General Circulation Model under the A1B emissions scenario. Panels on the top are based on projected environmental variables in the year 2030 and on the bottom for year 2070. The left panels are projections based on models built with the full dataset and panels on the right are based on models built with the invasive only dataset. The complementary log-log predicted probability for each raster cell is indicated by the color corresponding the bars to the right of the panels.

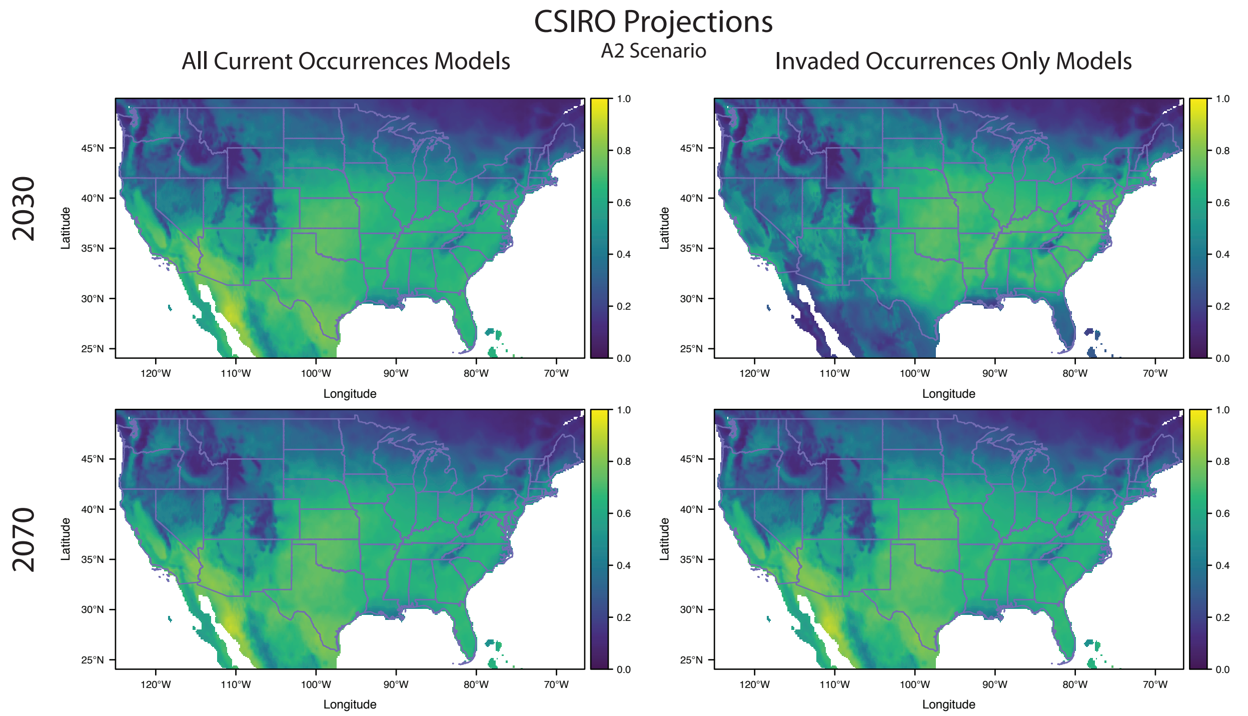

Figure S7. Model projection of *A. palmeri* under future climate change scenarios. The projections are for the CSIRO General Circulation Model under the A2 emissions scenario. Panels on the top are based on projected environmental variables in the year 2030 and on the bottom for year 2070. The left panels are projections based on models built with the full dataset and panels on the right are based on models built with the invasive only dataset. The complementary log-log predicted probability for each raster cell is indicated by the color corresponding the bars to the right of the panels.

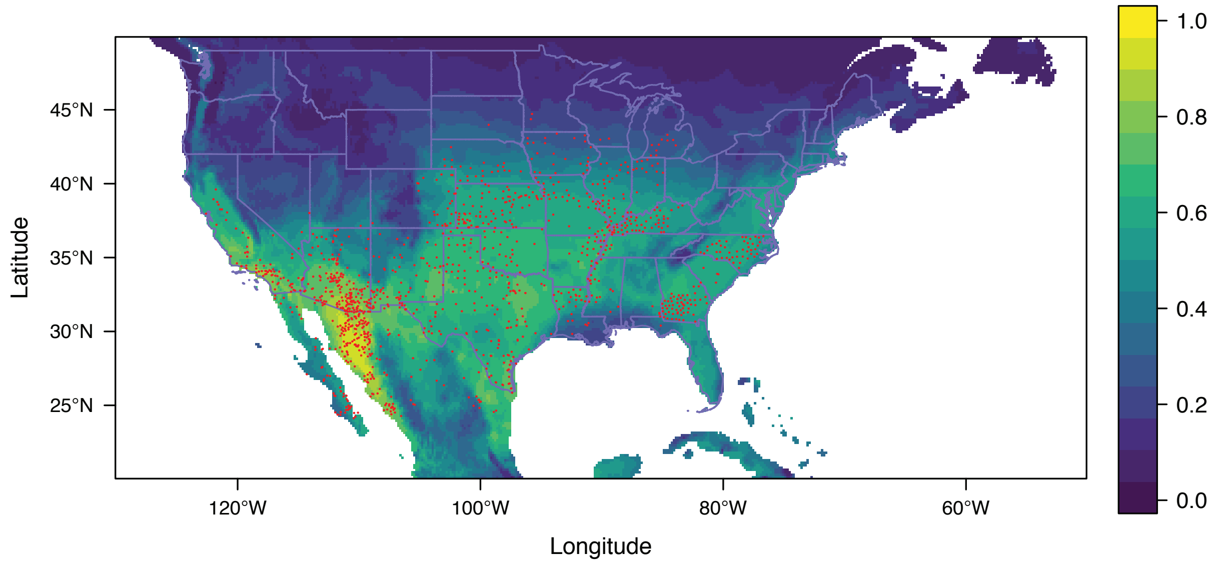

Figure S8. SDM model projection of *Amaranthus palmeri* generated using CliMond variables for the filtered dataset. The projection is the mean of 25 model runs. The occurrences included in the model are shown in red. The complementary log-log predicted probability for each raster cell is indicated by the color corresponding the bars to the right of the panel.

BOOSTED REGRESSION TREES

SUPPLEMENTARY METHODS & RESULTS

METHODS

Modeling Algorithm

To provide further validation of our Palmer Amaranth models, we built SDMs using Generalized Boosting Models (GBM; also known as Boosted Regression Trees (BRT)) for the dataset using all current occurrence records and for the invasive only dataset. BRT is a regression technique that combines many relatively simple regression tree models adaptively to optimize predictive power as opposed to building one 'best' model (Elith et al. 2008). BRT, along with Maxent, generally outperform other methods and has been shown to perform reliability in various modeling scenarios (Elith et al. 2008, Merow et al. 2013). We performed all modeling implementing functions in the 'gbm' and 'dismo' packages in R version 3.4.1.

Sampling pseudo-absences

In BRT, background sampling represents pseudo-absence and therefore we excluded presence points as potential pseudo-absence points by eliminating sampling from 5km buffers around presence points. For BRT models, the number of pseudo-absences was equal to approximately the number of presence points.

Boosted Regression Tree Model Building

We selected model parameters based on practices outlined in Elith et al. (2008). We optimized our learning rate using the 'gbm.step' function. We chose a learning rate of 0.001 using optimization of deviance (Friedman 2011, Elith et al 2008). This is the learning rate at

which deviance is minimized and where learning is sufficiently slow such that models with the lowest deviance contained at least 1000 trees and generally between 1000-2500 trees. We built models using tree complexity values of 2 and 5 (number of nodes allowed in the tree), to compare the results of simple models with no interactions between environmental variables or complex models with climate variable interactions. For all models, we used a bag fraction of 0.7. We overparameterized preliminary BRT models by using all available climatic variables, then simplified these models by implementing automatic bootstrapping and variable removal using the 'gbm.simplify' function and refitting a reduced model. We built a total of five models with five-fold cross validation to assess variability. We then projected the simplified models using the 'predict' function in 'dismo', assessed variable contributions, and evaluated model fit using AUC (averaged across cross validation datasets in model building, AUC-cv, and evaluated on a separate withheld testing dataset, AUC-test) and TSS.

RESULTS

All Available Occurrence Records

BRT models built using all current occurrence records had broadly similar geographical patterns of predicted suitability with models built using the MaxEnt algorithm (Figures 2, S9 &S10). Models built with both levels of tree complexity (2 and 5) produced similar projections. For both the CliMond and PRISM environmental variables, areas predicted to have the greatest probability of occurrence similar to MaxEnt models include: the native range, the southeastern United States, the Central Valley of California, portions of New England, and the Midwestern United States. Projections using the PRISM data also similarly predict higher probabilities of occurrence in the Palouse Region of Washington/Idaho and the in metropolitan areas at

northern latitudes, such as the Minneapolis/St. Paul region. Additionally, and contrary to MaxEnt models, BRT models indicate similar predicted probabilities in the Upper Midwest along the expanding invasion front to the Central Midwest and Southeastern United States, which are areas already facing intense invasion pressure. However, BRT models also indicated less discrimination between areas of predicted occurrence. Geographically, predicted probabilities of occurrence are more even across all areas of North America including in the native range and almost no areas have predicted probability of occurrence of greater than 0.8 or less than 0.2.

Despite overall similar patterns of prediction, BRT models did not perform as well in model evaluations as MaxEnt models and overall model performance statistics were poor to fair. With regard to model complexity, simpler models modestly outperformed more complex models. For models built with CliMond variables, AUC-cv was 0.60 ± 0.005 (range: 0.59-0.62), AUC-test was 0.57 ± 0.022 (range: 0.52-0.62) for models of tree complexity 2. For models of tree complexity 5, AUC-cv was 0.59 ± 0.01 (range: 0.56-0.61), AUC-test was 0.54 ± 0.015 (range: 0.51-0.58). TSS was significantly lower at 0.16 ± 0.022 (range: 0.11-0.22) and 0.12 ± 0.03 (range: 0.07-0.18), for tree complexity 2 and 5 respectively. For models built with PRISM data, AUC-cv was 0.56 ± 0.009 (range: 0.53-0.59), AUC-test was 0.56 ± 0.022 (range: 0.40-0.62) for models of tree complexity 2. For models of tree complexity 5, AUC-cv was 0.56 ± 0.01 (range: 0.55-0.59), AUC-test was 0.57 ± 0.015 (range: 0.53-0.61). TSS was significantly lower at 0.17 ± 0.03 (range: 0.11-0.26) and 0.16 ± 0.02 (range: 0.11-0.21), for tree complexity 2 and 5 respectively.

BRT models were built using all available environmental variables and then simplified using bootstrapping and variable removal so the models all contain different subsets of variables. Among models built with CliMond variables, all models were heavily influenced by

temperature variables and secondarily precipitation and radiation variables (Table S3). Most models retained the variable for mean temperature of the warmest quarter and also generally retained mean temperature of the wettest quarter or mean temperature of the driest quarter. In some models, mean annual precipitation and radiation seasonality were also retained. For models built with PRISM variables, temperature and vapor pressure deficiency were included in nearly all models. Most models retained mean annual temperature as the variable with the greatest relative influence and included minimum and/or maximum vapor pressure deficiency (Table S3).

Invasive Occurrence Records

BRT models built using records from the invaded range project geographically similar areas of highest predicted probabilities of occurrence to models built using MaxEnt. These models have the greatest probabilities of occurrence in the southeastern United States, the Midwest, portions of New England, and the Central Valley of California (Figures S9 & S10). Additionally, the models built with PRISM environmental variables also indicate higher predicted probabilities in metropolitan areas at northern latitudes and in the Palouse region of Washington/Idaho. Similar to the models built with all available records, there is more even distribution of predicted probabilities with most predicted probabilities of occurrence falling between 0.2 and 0.8.

Model performance was generally poor to fair and simple models seemed to modestly outperform more complex models. For models built with CliMond variables, AUC-cv was 0.61 ± 0.009 (range: 0.59-0.62), AUC-test was 0.59 ± 0.02 (range: 0.53-0.62), and TSS was 0.19 ± 0.03 (range: 0.10-0.24) for models with tree complexity 2. AUC-cv was 0.58 ± 0.008 (range: 0.56-

0.60), AUC-test was 0.53 ± 0.01 (range: 0.50-0.56), and TSS was 0.14 ± 0.01 (range: 0.12-0.16) for models with tree complexity 5. For models built with PRISM variables and models with tree complexity 2, AUC-cv was 0.60 ± 0.008 (range: 0.58-0.62), AUC-test was 0.59 ± 0.02 (range: 0.53-0.61), and TSS was 0.19 ± 0.03 (range: 0.10-0.24). For models with tree complexity 5, AUC-cv was 0.59 ± 0.01 (range: 0.55-0.61), AUC-test was 0.58 ± 0.01 (range: 0.53-0.60), and TSS was 0.18 ± 0.02 (range: 0.14-0.24).

Temperature variables had the greatest relative influence in BRT models built with invasive occurrence records. In models using CliMond variables, mean temperature of the warmest quarter was included in the majority of models and when it was not included mean annual temperature, mean temperature of the wettest quarter, or radiation of the coldest quarter were generally included. When PRISM data was used, mean or minimum temperature were in all models followed by minimum vapor pressure deficiency (Table S3).

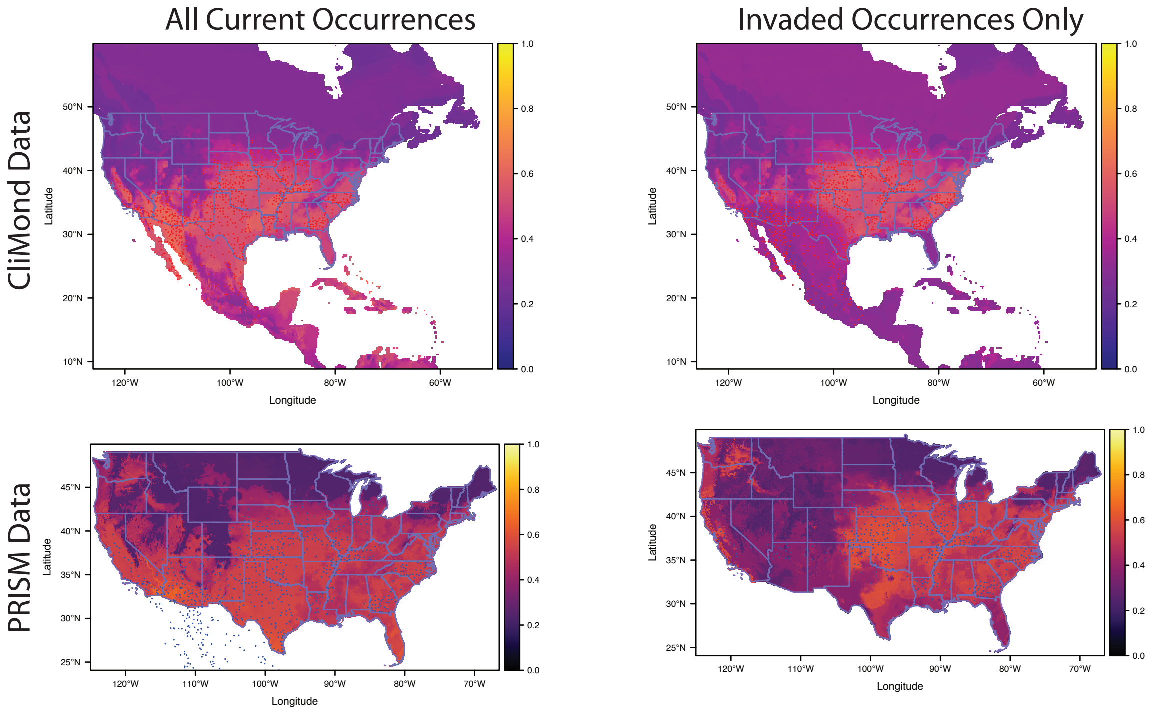

Figure S9. Boosted Regression Tree model with tree complexity equal to 2 (no variable interactions) projections of *Amaranthus palmeri* generated using CliMond and PRISM variables for the full dataset and invasive only dataset. All projections are means of 25 model runs. Top panels were generated using CliMond environmental variables and bottom panels were generated using PRISM variables. Panels on the left used the full dataset for model building and panels on the right used the invasive only dataset. In top panels the occurrences included in the model are shown in red and in the bottom panels the occurrences included in the model are shown in blue. All other occurrences are in gray. The complementary log-log predicted probability for each raster cell is indicated by the color corresponding the bars to the right of the panels.

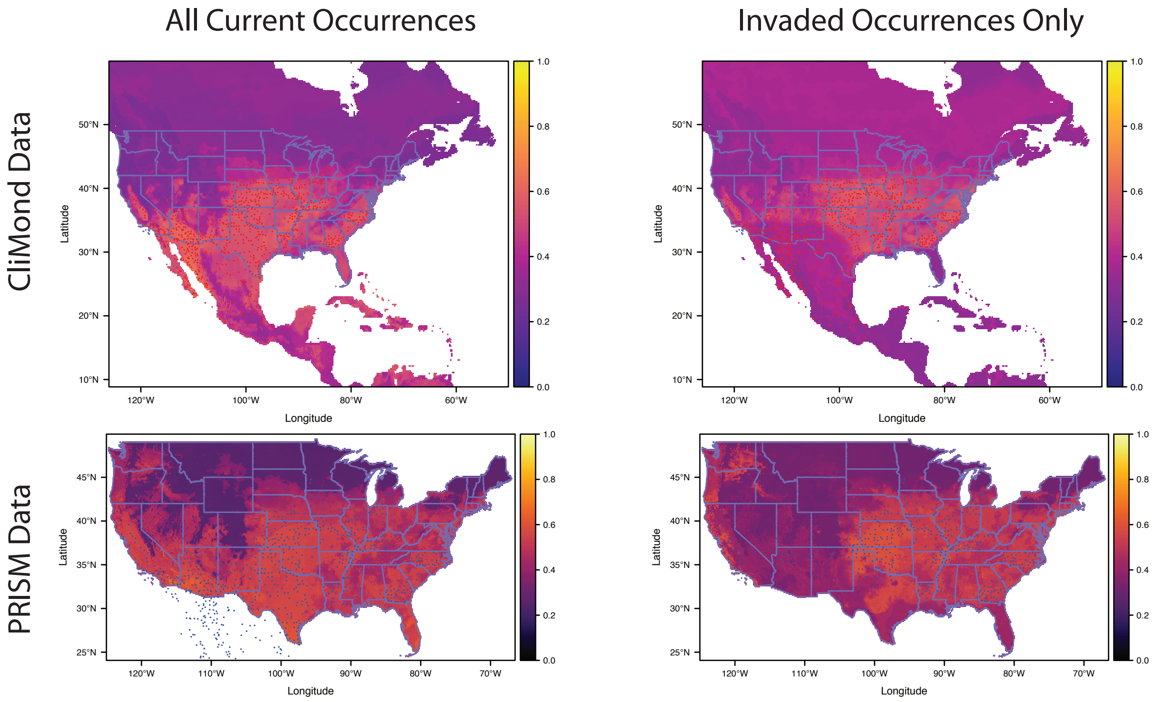

Figure S10. Boosted Regression Tree model with tree complexity equal to 5 (variable interactions possible) projections of *Amaranthus palmeri* generated using CliMond and PRISM variables for the full dataset and invasive only dataset. All projections are means of 25 model runs. Top panels were generated using CliMond environmental variables and bottom panels were generated using PRISM variables. Panels on the left used the full dataset for model building and panels on the right used the invasive only dataset. In top panels the occurrences included in the model are shown in red and in the bottom panels the occurrences included in the model are shown in blue. All other occurrences are in gray. The complementary log-log predicted probability for each raster cell is indicated by the color corresponding the bars to the right of the panels.

Table S3. Boosted regression tree model's environmental variable relative importance. Models were built with all available environmental data (CliMond or PRISM) and then simplified. Variable importance's are reported for 5 model runs based on 5-fold cross validation. Blank spaces indicate that the variable was not included in the final model.

		Native + invaded Dataset										Invaded Dataset									
		Relative Importance										Relative Importance									
		TC2					TC5					TC2					TC5				
**CliMond**		Model 1	Model 2	Model 3	Model 4	Model 5	Model 1	Model 2	Model 3	Model 4	Model 5	Model 1	Model 2	Model 3	Model 4	Model 5	Model 1	Model 2	Model 3	Model 4	Model 5
Mean temperature of wettest quarter	bio1		13.3			11.4		15.9			21.8	27.1			24.5		32.5				
Mean diurnal temperature range	bio2								18			11.4			21.6		14.6		52		
Maximum temperature of the warmest week	bio5							14.2													
Minimum temperature of the coldest week	bio6																	34.9			
Mean temperature of wettest quarter	bio8		13.5	19.6		13.9		15.9	17.4	27.1	19.7				24.8	54				45	55.8
Mean temperature of driest quarter	bio9	25.8	20.8		24		23.7	20.1													
Mean Temperature of the Warmest Quarter	bio10	40.4	14.5	39.9	46.2	23.4	27.8		27.5	43.9	29.3	12.2	50.7	49.9			17.4	31.2			
Annual precipitation	bio12		12.1			17.5		18.7			29.3										
Precipitation of the wettest quarter	bio16	33.8	15	18.3			25.2					14.2	49.3				20	33.9			
Precipitation of the coldest quarter	bio19					9.1	23.3					11.1									
Annual mean radiation	bio20			22.2					18.6												
Radiation Seasonality	bio23		10.9		29.8	8.6		15.2		29											
Radiation of the wettest quarter	bio24					8.6															
Radiation of coldest quarter	bio27											11.8		50.1	29.1	46	15.5		48	55	44.2
Annual mean moisture index	bio28					7.5			18.5			12									
**PRISM**		Model 1	Model 2	Model 3	Model 4	Model 5	Model 1	Model 2	Model 3	Model 4	Model 5	Model 1	Model 2	Model 3	Model 4	Model 5	Model 1	Model 2	Model 3	Model 4	Model 5
Mean temperature		24.6	61.2	35.7	45.5	58.7		39.9	61.1	29.1	37		54.4	42.3				56.7	42.3		
Minimum temperature				17.3			23.7					46.3			52.2	53.7	49.9			51.1	
Maximum temperature		22.2			31.8	41.3	27			18.7	28.9										
Precipitation		15.2		15.6						19.6	34.1										48
Minimum vapor pressure deficiency		23.3		17.3	22.7		30.1	27.5	38.9	15.5		53.7	45.6	57.7	47.8	46.3	50.1	43.3	57.7	48.9	52
Maximum vapor pressure deficiency			38.8	14			19.2	32.6		17											
Dew Point		14.7																			

REFERENCES

Elith J, Leathwick JR, Hastie T (2008) A working guide to boosted regression trees. J Anim Ecol, 77: 802-813.

Merow C, Smith MJ, Silander JA (2013) A practical guide to MaxEnt for modeling species distributions: what it does, and why inputs and settings matter. Ecography, 36: 1058-1069.

Appendix A: Occurrence Record Documentation

Database	Date	Number of Records	Number of datasets	DOI/reference
GBIF	18-Feb-17	871	75	http://doi.org/10.15468/dl.avol1h
GBIF	30-Jun-16	1790	99	http://doi.org/10.15468/dl.nuk8tm
GBIF	30-Jun-16	1790	99	http://doi.org/10.15468/dl.47qgqc
GBIF	30-Jun-16	1790	99	http://doi.org/10.15468/dl.yvwslu
EDDMaps	18-Feb-17	120	1	http://bugwoodcloud.org/eddmaps/csv/6694.csv
SEINET	11-Jul-17	2392	1	http//:swbiodiversity.org/seinet/index.php
Land Manager Communication	18-Feb-17	434	12	County-level records
